# Effect of Renal Ischemia Reperfusion on Brain Neuroinflammation

**DOI:** 10.3390/biomedicines10112993

**Published:** 2022-11-21

**Authors:** Bina Lee, Ingabire Ines, Jihyun Je, Eun Jung Park, Hyemin Seong, Min Gi Jo, Hwajin Kim, Seon-Hee Kim, Seong Jae Kim, Hye Jung Kim, Minkyeong Kim, Sang Won Park, Seung Pil Yun

**Affiliations:** 1 Department of Pharmacology, Institute of Health Sciences, College of Medicine, Gyeongsang National University, Jinju 52727, Republic of Korea; 2 Department of Ophthalmology, Institute of Health Sciences, College of Medicine, Gyeongsang National University, Gyeongsang National University Hospital, Jinju 52727, Republic of Korea; 3 Department of Pharmacology and Convergence Medical Science, Institute of Health Sciences, College of Medicine, Gyeongsang National University, Jinju 52727, Republic of Korea; 4 Department of Neurology, Gyeongsang National University Hospital, Jinju 52727, Republic of Korea

**Keywords:** renal ischemia reperfusion (IR), acute kidney injury (AKI), central nervous system (CNS), neuro-inflammation, neuronal toxicity

## Abstract

Acute kidney injury (AKI) is an inflammatory sequence. It can lead to distant organ injury, including damage to the central nervous system (CNS), mediated by increased circulating cytokines and other inflammatory mediators. It can also lead to increased blood–brain barrier (BBB) permeability. However, the effect of AKI on the inflammatory response of the brain has not yet been investigated. Therefore, we observed the effect of AKI on BBB permeability, microglia and astrocyte activation, and neuronal toxicity in the brain. The striatum and ventral midbrain, known to control overall movement, secrete the neurotransmitter dopamine. The activation of microglia and astrocytes present in this area causes neuro-degenerative diseases, such as Alzheimer’s disease (AD) and Parkinson’s disease (PD). The activation of astrocytes and microglia in the hippocampus and cerebral cortex, which are responsible for important functions, including memory, learning, concentration, and language, can trigger nerve cell apoptosis. The activation of astrocytes and microglia at this site is also involved in the inflammatory response associated with the accumulation of beta-amyloid. In the situation of kidney ischemia reperfusion (IR)-induced AKI, activation of microglia and astrocytes were observed in the striatum, ventral midbrain, hippocampus, and cortex. However, neuronal cell death was not observed until 48 h.

## 1. Introduction 

Kidney ischemia reperfusion (IR) is the major cause of acute kidney injury (AKI). It is considered a risk factor for incident chronic kidney disease, end-stage renal disease, hypertension, cardiovascular disease, and death in adults [1,2,3,4,5,6,7]. AKI is the consequence of renal blood flow interruption, urine flow obstruction, and the accumulation of toxins, which activate the immune system, leading to inflammation [1]. AKI is an inflammatory sequence that affects distant organs, leading to cardiovascular, lung, intestine, and liver injury [1,2,3,4,5,6,7]. Generally, AKI induces hypervolemia, the organ barrier function is disrupted, vascular permeability increases, and edema and the evolution of multiple microhemorrhages occurs [8,9,10]. Similarly, AKI increases the circulating of cytokines and other inflammatory mediators, due to increased production and reduced clearance, leading to increased blood–brain barrier (BBB) permeability and changes in neuronal homeostasis in the central nervous system (CNS) [11,12]. In spite of kidney IR being associated with various diseases, studies on the effect of kidney IR conditions on the brain, and especially on acute brain neuroinflammation, are rare. 

We hypothesized that AKI affects neurons and neuronal immune cells in the CNS. Generally, neurons transmit signals through neurotransmitters, and microglia are resident cells in the brain that regulate brain development, the maintenance of neuronal networks, and injury repair [13,14]. Glial cells, which are located between blood vessels and neurons, are known to be responsible for supporting nerve cells, supplying nutrients, removing waste, and phagocytosis [15]. The sustained activation of microglia and astrocytes influences the development and progression of neurodegenerative diseases, such as ischemia, seizures, Alzheimer’s disease, multiple sclerosis, amyotrophic lateral sclerosis, Parkinson’s disease (PD), and manganese disease [16,17,18,19,20]. Therefore, in this study, the effect of kidney IR conditions on brain neuroinflammation was investigated in an animal model.

As a result, we found that AKI disrupts the BBB and activates microglia and astrocytes throughout the brain. However, we did not observe the neuronal cell death induced by AKI in this study. In conclusion, AKI releases cytokines by disrupting the BBB. Microglia and astrocytes were activated throughout the brain, triggering brain inflammation-related factors. This suggests that AKI can sufficiently affect the brain and has the potential to promote the onset and exacerbation of neurodegenerative diseases.

## 2. Materials and Methods

### 2.1. Experimental Animals

Seven-week-old male C57BL/6 mice were obtained from Koatech (Pyeongtaek, South Korea) and maintained in the animal facility at Gyeongsang National University (GNU). All animal experiments were approved by the Institutional Board of Animal Research at GNU (GNU-200612-M0036) and performed in accordance with the National Institutes of Health guidelines for laboratory animal care. Mice were housed with alternating 12 h light/dark cycle with free access to food and water.

### 2.2. Animal Model of Kidney Ischemia Reperfusion

Mice were divided into four groups: (1) sham-operated mice (n = 21); (2) mice subjected to bilateral kidney IR and sacrificed 24 h after reperfusion (n = 21); (3) mice subjected to bilateral kidney IR and sacrificed 48 h after reperfusion (n = 21). We used three mice for Western blot, three mice for IF, three mice for IHC, six mice for mRNA, three mice for BBB disruption, and three mice for TUNEL assay. The mice were anesthetized with zoletil (0.5 mg/kg; Virbac Laboratories, Carros, France) intraperitoneally (I.P) and placed horizontally on a heating pad under a heat spotlight to maintain body temperature at 37 °C through the surgical procedures. Kidneys were exposed, and both renal pedicles were clamped for 25 min with micro-aneurysm clips. After 25 min of ischemia, the clips were removed to allow for reperfusion, and the abdomen was closed by suture [21]. Mice were sacrificed 24 h and 48 h after reperfusion, and then brains were removed, washed in PBS over an ice surface, dissected, and snap frozen in liquid nitrogen for storage at −80 °C for further analysis.

### 2.3. In Vivo BBB Permeability Measurements

To assess the in vivo permeabilization of the BBB in the kidney IR mouse model, a modified Evan’s blue dye assay was performed. Mice were anesthetized with zoletil (0.5 mg/kg; Virbac Laboratories, Carros, France) intraperitoneally (I.P). Evan’s blue dye was injected (5 mg/mL; 200 μL) and allowed to circulate for 20 min, at which time, mice were killed. Brains were removed and sliced. Sliced brain tissues were immersed in 3 mL of formamide and then incubated at 60 °C for 48 h to extract the dye. The concentration of the dye was similarly determined spectrophotometrically (620 nm). 

### 2.4. Immunohistochemistry (IHC)

Brain tissues were perfused with PBS and kept in 4% PFA for 24 h. Then, they were moved to 30% sucrose for 2 days and, finally, were embedded in O.C.T (optimal cutting temperature) compound (Sakura Finetek Inc., Torrance, CA, USA). The brain tissues were dissected into 40 µm cryostat-thick frozen sections at −20 °C with a cryostat machine. Immunohistochemistry images were captured by using a BX61VS microscope (Olympus, Tokyo, Japan). IHC was performed on 40 μm thick serial brain sections. Frozen sections were perfused with blocking solution for 30 min at room temperature (RT); blocking solution comprised: 0.2% triton (Biosesang, Gyeonggi, republic of Korea), 0.02% sodium azide (Sigma-Aldrich, St. Louis, MO, USA), and normal goat serum (Jackson Immunoresearch Laboratories, Inc., Baltimore, MD, USA). In order to study gliosis, sections were incubated overnight at 4 °C with anti-glial fibrillary acidic protein (GFAP, MAB360, Millipore, Temecula, CA, USA, 1:2000) and anti-Iba1 (019-19741, Fujifilm Wako Chemicals, Richmond, VA, USA, 1:1000). Sections were washed with TBST and incubated with a biotinylated secondary antibody (Vector Laboratories) for 1 h at RT. Sections were again washed and incubated in ABC reagents (Vector Laboratories, Inc., Burlingame, CA, USA), and then sections were developed using Sigma Fast (a 3,30 diaminobenzidine (DAB) Peroxidase Substrate Kit (Vector Laboratories)) and analyzed using a BX61VS microscope (Olympus, Tokyo, Japan). 

### 2.5. Immunofluorescence 

Histological assessments were performed on fixed frozen section of 40 μm thick serial brain sections with a cryostat, as described previously. Briefly, sections were permeabilized in 0.2% Triton X-100 (Biosesang) in PBS for 10 min at RT. The sections were incubated with the labeling reaction mixture at 37 °C for 60 min in a dark humidified chamber, followed by washing with PBS, fresh anti-tyrosine hydroxylase (TH, NB300-109, Novus biologicals, Centennial, CO, USA), and anti-class III beta-tubulin (TUJ-1, PRB-435P, Biolegend, San Diego, CA, USA) antibodies in dilution buffer (0.01 M PBS containing 5% normal goat serum and 0.2% Triton X 100) for immunofluorescence staining. Images were captured using an Olympus Fluoview FV1000 confocal microscope (Olympus, Tokyo, Japan). 

### 2.6. Terminal Deoxynucleotidyl Transferase dUTP Nick-End Labelling (TUNEL) Assay

TUNEL staining was performed to examine the degree of apoptosis using an In Situ Cell Death Fluorescein Detection Kit (Roche Molecular Biochemicals, Mannheim, Germany), according to the manufacturer’s instructions. The images were captured using an Olympus Fluoview FV1000 confocal microscopy (Tokyo, Japan).

### 2.7. Western Blot Analysis

Brain tissues were homogenized in ice-cold RIPA buffer (Thermo Fisher Scientific, Waltham, MA, USA) containing protease inhibitors (Thermo Fisher Scientific), sonicated, incubated on ice, and centrifuged for 20 min at 16,022× *g* at 4 °C. After centrifugation, the supernatant was collected, and the protein concentration was determined using the BCA protein assay kit (Thermo Fisher Scientific). The protein samples were electrophoresed on polyacrylamide gels and transferred to nitrocellulose membranes. After blocking, the membranes were incubated with the primary antibodies anti-TH (NB300-109, Novus biologicals, Centennial, CO, USA), anti-Iba-1 (016-20001 Fujifilm Wako Chemicals, Richmond, VA, USA), anti-GFAP (MAB360, EMD Millipore, Temecula, CA, USA), and anti-TUJ-1 (PRB-435P, Biolegend, San Diego, CA, USA) at 4 °C overnight. The membranes were incubated with horseradish peroxidase-conjugated secondary antibodies (Bio-Rad) at RT for 1 h. The membranes were incubated with electrochemiluminescence (ECL) substrates (Bio-Rad), and protein bands were visualized using the ChemiDoc XRS+ System (Bio-Rad).

### 2.8. Reverse Transcription and Quantitative PCR (qPCR) Analysis

Total RNA was extracted using Trizol (Invitrogen). RNA concentration was measured spectrophotometrically using a nanodrop (DeNvix Inc., Wilmington, CA, USA). Subsequently, 1–2 μg of total RNA was reverse transcribed to cDNA using a HiSecriptTM RH RT Premix Kit (Intron Biotechnology, Gyeonggi, Republic of Korea). Quantitative PCR was performed on the CFX Connect Real-Time PCR System using iQ SYBR Green Supermix (Bio-Rad). Relative mRNA levels were normalized to those of GAPDH for each gene. The primer sequences are listed in Table 1.

### 2.9. Single Microglia Separate Using CD1b+ Beads Kit

The sliced brain tissues was incubated in 0.25% trypsin (Sigma) in a 37 °C water bath for more than 15 min. Trypsinization was stopped by adding an equal volume EasySep™ Buffer (Catalog #20144). A single cell suspension of the brain tissue was prepared by gentle trituration and passed through a 100 μm nylon mesh cell strainer to remove tissue debris and aggregates. The single microglia were separated using EasySep™ Mouse CD11b Positive Selection Kit II (Catalog #18970, StemCell Technologies, Vancouver, BC, Canada). Briefly, the single cells mixed with microglia and astrocyte were contained in 5 mL (12 × 75 mm) polystyrene round-bottom tubes. Cell concentration was 2.5 × 10^7^ cells/mL. The cells were incubated with component A and component B mixture for 5 min. Then, we added Vortex RapidSpheres™ to single cells sample. The cells were incubated for 5 min. EasySep™ Buffer was added top up 2.5 mL, and tubes were placed into EasySep™ magnet (Catalog # 18000) and incubated for 5min. The supernatants were discarded. Buffer was added top up 2.5 mL, and magnet step was repeated more than three times. 

### 2.10. Immunofluorescence (IF) Analysis

Brain tissues were perfused with PBS and kept in the 4% PFA for 24 h, then moved to 30% sucrose for 2 days, finally, brains were embedded in O.C.T (optimal cutting temperature) compound (Sakura Finetek Inc., Torrance, CA, USA). The brain tissues were dissected into forty µm (40 μm) pieces, cryostat-thick frozen sections at −20 ℃, with a cryostat machine. The tissues were blocked with blocking buffer (0.2% triton (Biosesang, Gyeonggi, Korea), 0.02% sodium azide (Sigma-Aldrich) normal goat serum (Jackson Immunoresearch Laboratories, Inc., Baltimore, MD, USA). Primary and secondary antibodies and working dilutions were 1:500 ratio. The amounts of positive intensity were measured with ImageJ software. 

### 2.11. Statistical Analysis

All data were analyzed using the GraphPad Prism 7 software (Graph Pad Software, San Diego, CA, USA). Comparisons between groups were conducted by one-way ANOVA. Dunnett’s multiple comparison test was used to assess the statistical significance. Data are expressed as mean ± S.E.M with *p* < 0.05 were considered significant. More details are shown in Supplementary Data S1.

## 3. Results

### 3.1. Kidney IR Injury Increases the Permeability of BBB

The blood–brain barrier (BBB) has the main role of protecting the brain from harmful substances brought by the blood, in order to maintain the homeostasis of the central nervous system (CNS). In normal physiology, the permeability of the BBB is highly maintained. Nevertheless, in some pathophysiological conditions, such as injury, stroke, and trauma, vascular permeability increases, which leads to the disruption of the BBB [22]. 

**Figure 1:**
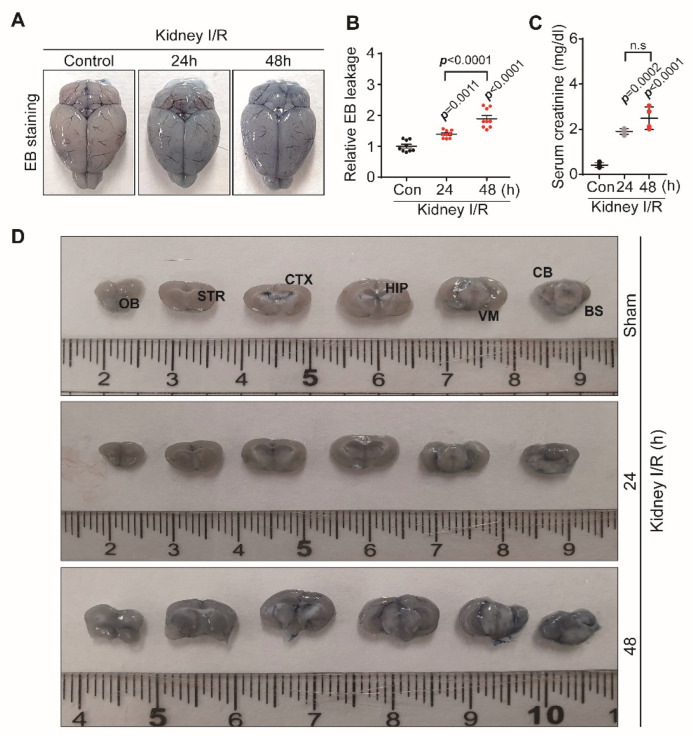
Kidney IR injury impaired BBB permeability. C57BL/6 mice sham (control) were operated, subjected to 25 min bilateral kidney IR, and sacrificed at 24 h and 48 h after reperfusion. (**A**) Analysis of Evans blue (EB) stain extravasation into the brain of mice from each group. (**B**) Relative EB leakage levels in the brain samples. (**C**) Plasma creatinine level**.** (**D**) EB staining on different regions of the brain. OB: olfactory brain; STR: striatum; CTX: cortex; HIP: hippocampus; VM: ventral midbrain; CB: cerebellum; BS: brain stem after brain dissection. The data represent the mean ± S.E.M for mice from each group; *p* < 0.005 was significant, as compared with the sham group.

To assess the role of kidney IR injury in BBB permeability, samples from sham mice and mice subjected to 25 min of bilateral kidney IR, which were sacrificed 24 h and 48 h after reperfusion, were stained with Evans blue (EB) dye. Then, EB stain extravasation from the above-mentioned groups was analyzed (Figure 1A,B,D). The results from this study demonstrate a huge increase in BBB permeability in the ischemic area of mice sacrificed at 24 h and 48 h after reperfusion. Serum creatinine was measured to confirm that the AKI model was successfully constructed. Serum creatinine was significantly increased, as compared to the control, at 24 h and 48 h after reperfusion (Figure 1C).

### 3.2. Kidney IR Injury Induced Microglia and A1 Astrocyte Activation in Striatum and VM 

**Figure 2:**
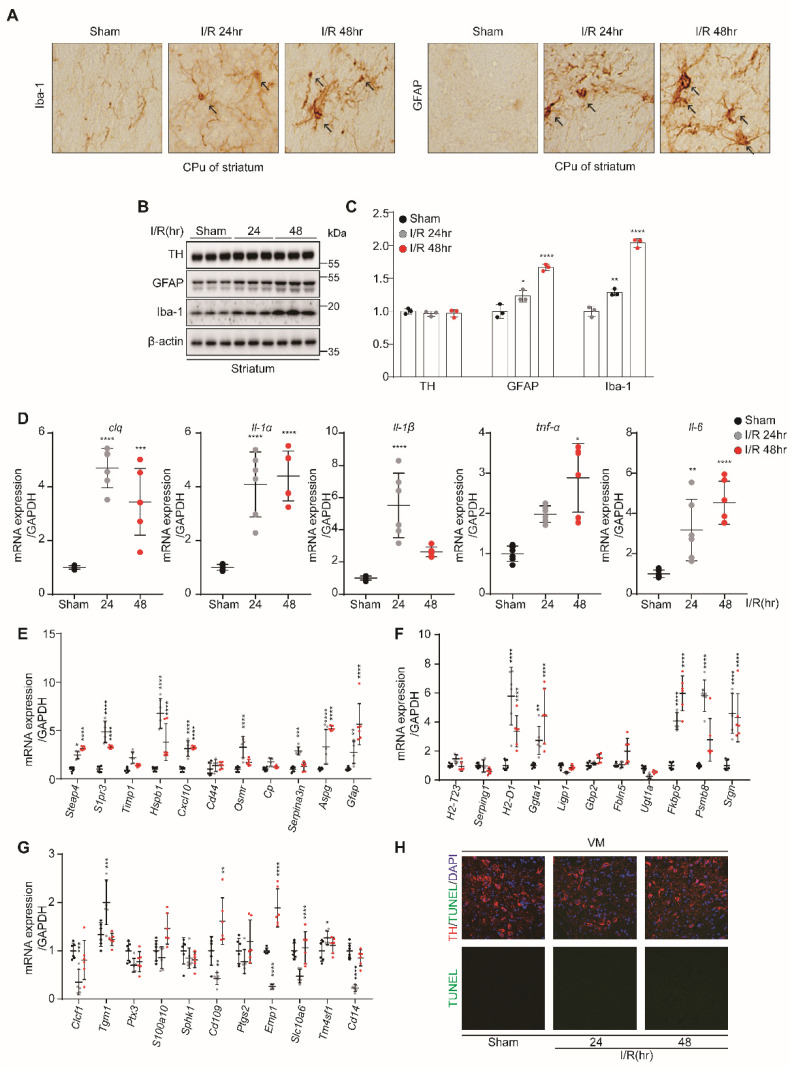
Kidney IR injury activates the microglia and astrocytes in the striatum region without affecting dopamine neuronal cell death. C57BL/6 mice sham (control) were operated, subjected to 25 min bilateral kidney IR, and sacrificed at 24 and 48 h after reperfusion. (**A**) Frozen striatum brain tissues were dissected and then incubated with anti-Iba-1 and anti-GFAP antibodies for IHC staining (n = 3, biologically independent animals). (**B**) The protein expressions of TH, GFAP, and Iba-1 were examined in the striatum brain tissue lysates using Western blot analysis (n = 3, biologically independent animals). (**C**) The relative protein level quantification of band densities. (**D**) The expressions of inflammatory mediators (*C1q*, *Il1a*, *Il1b*, *Tnfa,* and *Il6*) were determined using quantitative qPCR analysis in RNA samples of stratum brain tissues. (**E**–**G**) The gene markers of astrocytes, i.e., PAN-reactive transcripts, A1-specific transcripts, and A2-specific transcripts, were examined using quantitative PCR analysis in striatum RNA samples. Values are represented as mean ± S.E.M of independent mice. * *p* < 0.05, ** *p* < 0.01, *** *p* < 0.001, **** *p* < 0.0001 were significant, as compared with the sham group. (**H**) Apoptosis of dopamine neurons in the striatum was determined by TUNEL assay (green); TH is shown in red.

To determine whether kidney IR induces microglia and astrocyte activation in vivo, Iba-1 and GFAP were assessed in striatum tissues using IHC. Kidney IR injury induces the high expressions of GFAP (astrocytes markers) and Iba-1 (microglia marker) in mice subjected to kidney IR and sacrificed at 24 h and 48 h after reperfusion (Figure 2A). This effect was confirmed by Western blot analysis, in which the expression level of GFAP and Iba-1 were increased at 24 h and 48 h, following kidney IR injury. However, TH, as a marker of dopamine neurons, was not affected (Figure 2B,C). The findings of this study show that kidney IR induces microglia and astrocyte activation without affecting the dopamine neurons. 

To further investigate the ability of kidney IR to activate microglia, the mRNA levels of *C1q, Il1a, Il1b, Tnfa,* and *Il6* were determined using qPCR in striatum tissues and kidneys. It was shown that IR injury significantly increased the expressions of these pro-inflammatory mediators in mice sacrificed at 24 h and 48 h after reperfusion.

To investigate the role of kidney IR in the induction of astrocyte reactivity by qPCR, the mRNA levels of PAN-transcripts (markers of general astrocyte reactive state), A1-specific transcripts, and A2-specific transcripts were evaluated. Kidney IR significantly increased the expressions of A1-specific transcripts and PAN-transcripts in mice sacrificed at 24 h and 48 h after reperfusion (Figure 2E,F). The resulting A1-reactive astrocytes secreted proteinaceous toxins that killed many types of neurons and oligodendrocytes [23]. Thus, A1-reactive astrocytes appear to exhibit deleterious properties that can damage neurons and oligodendrocytes in vivo [24]. A2-reactive astrocytes are proposed to be neuroprotective because they upregulate many neurotrophic factors that promote neuronal survival and tissue repair [23]. Gene subsets, described as PAN-reactive genes, include Gfap, Vim, and Cxcl10. These PAN-reactive genes are expressed by both A1- and A2-reactive phenotypes and appear to be universal markers of astrocytosis [23]. A2-specific transcripts were not affected by kidney IR injury in mice sacrificed at 24 h and 48 h after reperfusion. 

Finally, the effect of kidney IR on dopamine neuronal cell death in the striatum was determined by TUNEL assay in the striatum tissues in the three groups of mice. The results indicate the absence of neuronal cell death in all groups (Figure 2H). These results suggest the critical role of kidney IR in inducing the activation of microglia, leading to increased cytokine gene expression, which, in turn, mediates the increased activity of A1 astrocytes without causing dopamine neuronal cell death. 

### 3.3. Kidney IR Injury Induced Microglia and A1 Astrocyte Activation in the VM

**Figure 3:**
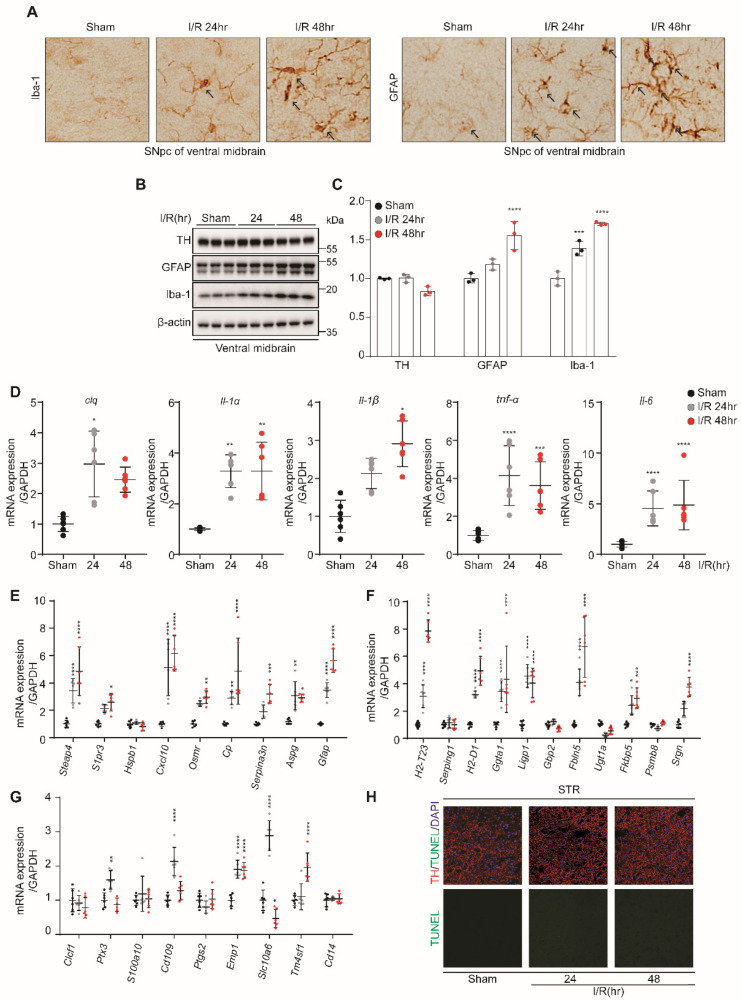
Kidney IR injury activates the microglia and astrocytes in ventral midbrain (VM) region without effecting dopamine neuronal cell death. C57BL/6 mice sham (control) were operated, subjected to 25 min bilateral kidney IR, and sacrificed at 24 h and 48 h after reperfusion. (**A**) Frozen VM brain tissues were dissected and then incubated with anti-Iba-1 and anti-GFAP antibodies for IHC staining (n = 3, biologically independent animals). (**B**) The protein expressions of TH, GFAP, and Iba-1 were examined in the VM tissue lysates using Western blot analysis (n = 3, biologically independent animals). (**C**) The relative protein level quantification of band densities. (**D**) The expressions of inflammatory mediators (*C1q*, *Il1a*, *Il1b*, *Tnfa,* and *Il6*) were determined using quantitative qPCR analysis in RNA samples of VM tissues. (**E**–**G**) The gene markers of astrocytes, i.e., PAN-reactive transcripts, A1-specific transcripts, and A2-specific transcripts, were examined using quantitative PCR analysis in VM RNA samples. Values are represented as mean ± S.E.M of independent mice. * *p* < 0.05, ** *p* < 0.01, *** *p* < 0.001, **** *p* < 0.0001 were significant, as compared with the sham group. (**H**) Apoptosis of dopaminergic neurons in the VM was determined by TUNEL assay (green); TH is shown in red.

The IHC analysis showed that kidney IR injury induces the high expression GFAP and Iba-1 in mice sacrificed at 24 h and 48 h after reperfusion (Figure 3A). This effect was confirmed by Western blot analysis, in which the expression levels of GFAP and Iba-1 were increased in mice sacrificed at 24 h and 48 h after reperfusion. However, TH, as a marker of dopamine neurons, was not affected (Figure 3B,C). The findings of this study show that kidney IR injury induces microglia and astrocyte activation without affecting the dopamine neurons. 

Once the microglia and astrocytes are activated, they upregulate certain genes that are involved in neuroinflammation. To further investigate the ability of kidney IR injury to activate the microglia, the mRNA levels of *C1q*, *Il1a*, *Il1b*, *Tnfa,* and *Il6* were determined using qPCR in VM tissues. The results showed that kidney IR injury significantly increased the expressions of the aforementioned inflammatory mediators in mice sacrificed at 24 h and 48 h after reperfusion (Figure 3D). 

To determine whether kidney IR injury induces reactive astrocytes, using qPCR, the mRNA levels of PAN-transcripts, A1-specific transcripts, and A2-specific transcripts were evaluated. Kidney IR significantly increased the expressions of A1-specific transcripts and PAN-transcripts in mice sacrificed at 24 h and 48 h after reperfusion (Figure 3E,F). The A2-specific transcripts were not affected by kidney IR in mice sacrificed at 24 h and 48 h after reperfusion.

Finally, the effect of kidney IR injury on dopamine neuron cell death in the VM was determined by performing the TUNEL assay on the VM tissues of mice. The results indicate the absence of dopamine neuronal cell death in all groups (Figure 3H). These results suggest the critical role of kidney IR injury in inducing the reactivation of microglia, leading to increased cytokine gene expression, which, in turn, mediates the increased activity of A1 astrocytes without causing dopamine neuronal cell death in the VM. 

### 3.4. Microglia and A1 Astrocytes Were Activated by Kidney IR Injury in the Hippocampus and Cortex

**Figure 4:**
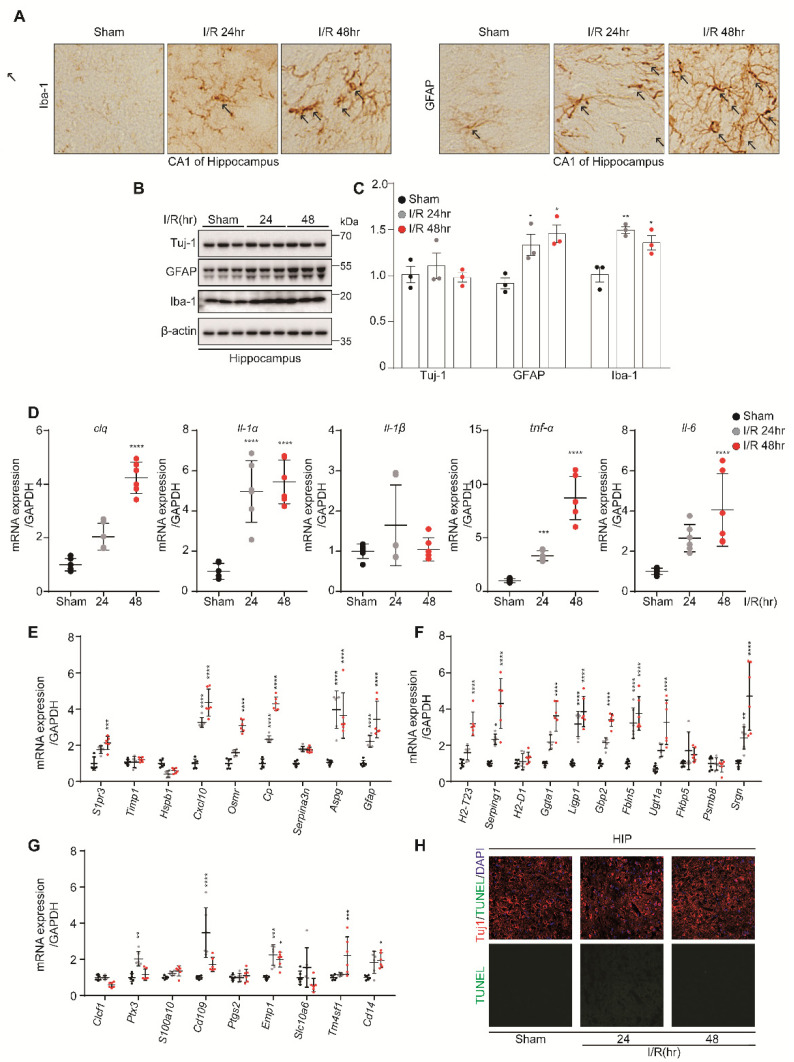
Kidney IR injury promotes the activation of microglia and astrocytes in the hippocampus region without effecting neuronal cell death. C57BL/6 mice sham (control) were operated, subjected to 25 min bilateral kidney IR, and sacrificed at 24 h and 48 h after reperfusion. (**A**) Frozen hippocampus brain tissues were dissected and then incubated with anti-Iba-1 and anti-GFAP antibodies for IHC staining (n = 3, biologically independent animals). (**B**) The protein expressions of TH, GFAP, and Iba-1 were examined in the hippocampus tissue lysates using Western blot analysis (n = 3, biologically independent animals). (**C**) The relative protein level quantification of band densities. (**D**) The expressions of inflammatory mediators (*C1q*, *Il1a*, *Il1b*, *Tnfα,* and *Il6*) were determined using quantitative qPCR analysis in RNA samples of VM tissues. (**E**–**G**) The gene markers of astrocytes, i.e., PAN-reactive transcripts, A1-specific transcripts, and A2-specific transcripts, were examined using quantitative PCR analysis in Hippocampus RNA samples. Values are represented as mean ± S.E.M of independent mice. * *p* < 0.05, ** *p* < 0.01, *** *p* < 0.001, **** *p* < 0.0001 were significant, as compared with the sham group. (**H**) Apoptosis of neurons in the hippocampus was determined by TUNEL assay (green); TUJ-1 is shown in red.

IHC analysis showed that kidney IR injury induced the high expressions of GFAP and Iba-1 in mice subjected to kidney IR and sacrificed at 24 h and 48 h after reperfusion (Figure 4A). This effect was confirmed by Western blot analysis, in which the expression levels of GFAP and Iba-1 were increased in mice sacrificed after at 24 h and 48 h of reperfusion. However, Tuj-1, as a marker of neurons, was not affected (Figure 4B,C). The findings of this study show that kidney IR injury induces microglia and astrocyte activation without harming neurons.

Once the microglia and astrocytes are activated, they upregulate genes that are implicated in neuroinflammation. To further investigate whether kidney IR activates the microglia, the mRNA levels of C1p, IL-1α, IL-1β, TNF-α, and IL-6 were determined in the hippocampus tissues using qPCR. It was shown that kidney IR injury significantly increased the expressions of these pro-inflammatory mediators in the mice sacrificed at 24 h and 48 h after reperfusion (Figure 4D).

To determine the role of kidney IR injury in inducing reactive astrocytes, using qPCR, the mRNA levels of PAN-transcripts, A1-specific transcripts, and A2-specific transcripts were assessed. Kidney IR significantly increased the expressions of A1-specific transcripts and PAN-transcripts in mice sacrificed at 24 h and 48 h after reperfusion (Figure 4E,F). A2-specific transcripts were not affected by kidney IR injury in mice sacrificed at 24 h and 48 h after reperfusion (Figure 4G).

Finally, the effect of kidney IR injury on neuron cell death was determined in hippocampus tissues of mice using the TUNEL assay. The results demonstrate the absence of neuronal cell death in all groups (Figure 4H). These results suggest the critical role of kidney IR in inducing the activation of the microglia, leading to increased pro-inflammatory mediator gene expression, which, in turn, mediates the increased activity of A1 astrocytes without causing neuronal cell death in the hippocampus region. 

### 3.5. Kidney IR Injury Induced Microglia Activation and A1 Astrocyte Formation in the Cortex

**Figure 5:**
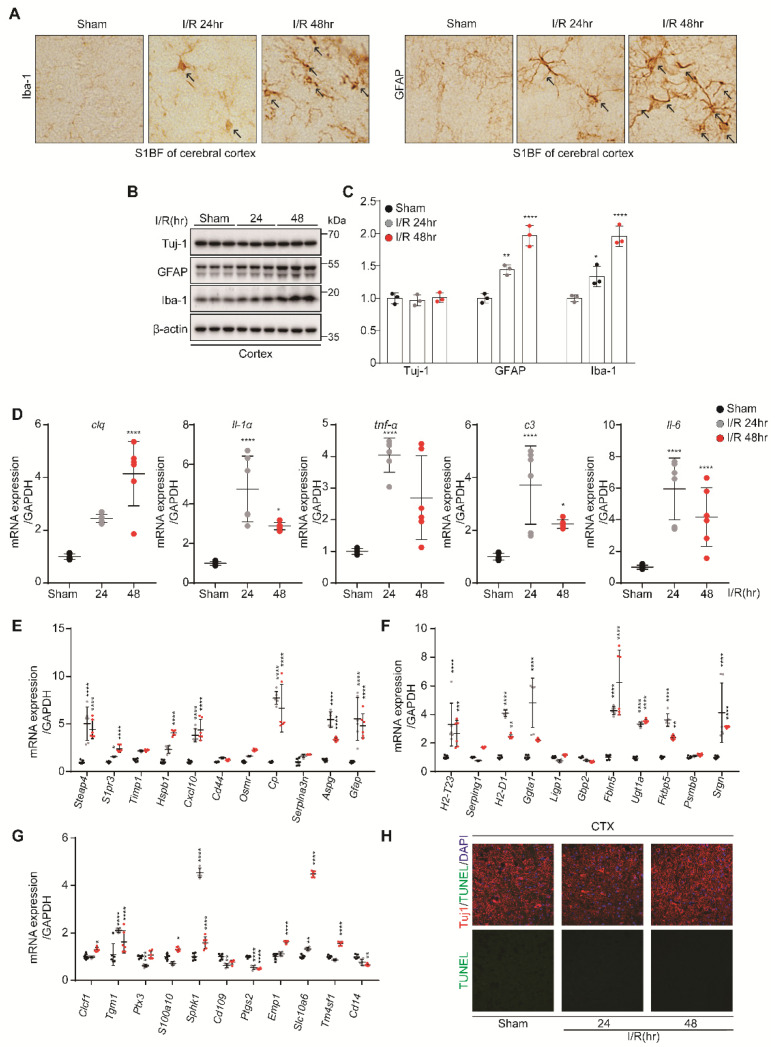
Kidney IR activates microglia and astrocytes in the cortex region without effecting neuronal death. C57BL/6 mice sham (control) were operated, subjected to 25 min bilateral kidney IR, and sacrificed at 24 h and 48 h after reperfusion. (**A**) Frozen cortex brain tissues were dissected and then incubated with anti-Iba-1 and anti-GFAP antibodies for immunohistochemistry staining (n = 3, biologically independent animals). (**B**) The protein expressions of TH, GFAP, and Iba-1 were examined in the cortex tissue lysates using Western blot analysis (n = 3, biologically independent animals). (**C**) The relative protein level quantification of band densities. (**D**) The expressions of inflammatory mediators (*C1q*, *Il1a*, *Il1b*, *Tnfa,* and *Il6*) were determined using qPCR analysis in RNA samples of cortex tissues. (**E**–**G**) The gene markers of astrocytes, i.e., PAN-reactive transcripts, A1-specific transcripts, and A2-specific transcripts, were examined using quantitative PCR analysis in cortex RNA samples. Values are represented as mean ± S.E.M of independent mice. * *p* < 0.05, ** *p* < 0.01, *** *p* < 0.001, **** *p* < 0.0001 were significant, as compared with the sham group. (**H**) Apoptosis of neurons in the cortex was determined by TUNEL assay (green); TUJ-1 is shown in red.

The IHC analysis showed that kidney IR injury induced the high expression GFAP and Iba-1 in mice sacrificed at 24 h and 48 h after reperfusion (Figure 5A). This effect was confirmed by Western blot analysis, in which the expression levels of GFAP and Iba-1 were increased in mice sacrificed after 24 h and 48 h. However, Tuj-1, as a marker of neurons, was not affected (Figure 5B,C). The findings of this study show that kidney IR injury induces microglia and astrocyte activation without killing neurons. 

Once the microglia and astrocytes are activated, they upregulate the genes that are implicated in neuroinflammation. To further investigate the ability of kidney IR to activate microglia, the mRNA levels of C1p, IL-1α, IL-1β, TNF-α, and IL-6 were determined in cortex tissues using qPCR. Kidney IR injury significantly increased the expressions of these pro-inflammatory mediators in mice sacrificed at 24 h and 48 h after reperfusion (Figure 5D). 

To determine whether kidney IR injury induced astrocyte reactivity, the mRNA levels of PAN-transcripts, A1-specific transcripts, and A2-specific transcripts were assessed by qPCR. Kidney IR injury significantly increased the expressions of A1-specific transcripts and PAN-transcripts in mice sacrificed at 24 h and 48 h after reperfusion (Figure 5E,F). A2-specific transcripts were not affected by kidney IR injury in mice sacrificed at 24 h and 48 h after reperfusion (Figure 5G).

Finally, the effect of kidney IR injury on neuronal cell death in the cortex was determined from the cortex tissues of mice using the TUNEL assay. The results indicate the absence of neuronal cell death in all groups (Figure 5H). These results suggest a critical role of kidney IR injury in inducing the activation of microglia, leading to increased pro-inflammatory mediator gene expression, which, in turn, mediates the increased activity of A1 astrocytes without causing neuronal cell death in the cortex region. Additionally, cytokines were observed in the pure microglia cells in STR, VM, HIP, and CTX regions. In microglia cells isolated with the CD11b positive selection kit, it was confirmed that the expressions of *C1q, Il1α, Il1b, Tnfα,* and *Il6* were increased in the 24 h and 48 h groups, compared to the Sham group (Supplementary Figure S1).

### 3.6. Kidney IR Induced Microglia Activation and A1 Astrocyte Formation in the Olfactory Bulb, Cerebellum, and Brain Stem

**Figure 6:**
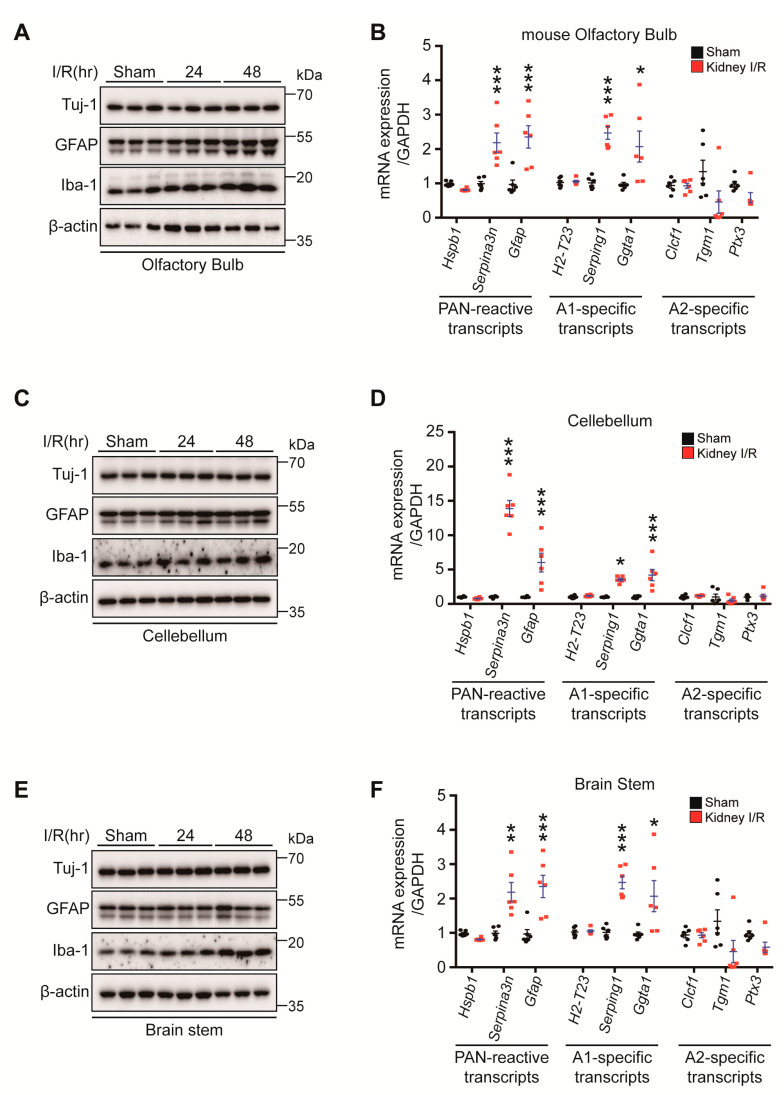
Kidney IR injury activates microglia and astrocytes in the olfactory bulb, cerebellum, and brain stem regions. C57BL/6 mice sham (control) were operated, subjected to 25 min bilateral kidney IR, and sacrificed at 48 h after reperfusion. (**A**) Protein expressions of Tuj-1, GFAP, and Iba-1 were determined in OB lysates by Western blot analysis for three different mice. (**B**) Astrocyte gene markers (PAN-reactive transcripts, A1-specific transcripts, and A2-specific transcripts) were determined using quantitative analysis of RNA samples in OB. (**C**) Protein expressions of Tuj-1, GFAP, and Iba-1 were determined in CB lysates by Western blot analysis for three different mice. (**D**) Astrocytes gene markers (PAN-reactive transcripts, A1-specific transcripts, and A2-specific transcripts) were determined using quantitative analysis of RNA samples in CB. (**E**) Protein expressions of Tuj-1, GFAP, and Iba-1 were determined in BS lysates by Western blot analysis for three different mice. (**F**) The gene markers of astrocytes, i.e., PAN-reactive transcripts, A1-specific transcripts, A2-specific transcripts, were determined using quantitative analysis of RNA samples in BS. Values are represented as mean ± S.E.M of independent mice. * *p* < 0.05, ** *p* < 0.01, *** *p* < 0.001 were significant, as compared with the sham group.

To examine whether kidney IR injury induces microglia and astrocyte activation in vivo, the Iba-1 and GFAP levels from seven-week-old male C57BL/6 mice subjected to bilateral kidney IR injury and sacrificed at 24 h and 48 h were analyzed. Western blot analysis showed that kidney IR injury induced the high expression GFAP and Iba-1 in mice sacrificed at 24 h and 48 h after reperfusion in the olfactory bulb and cerebellum, while, in the brain stem, only Iba-1 expression was increased at 48 h after reperfusion (Figure 6).

### 3.7. Colocalization of Iba-1 and Cytokines, Neutrophils and Cytokines in STR, VM, HIP and CTX

To investigate whether Kidney IR injury induces colocalization of activated microglial and the cytokines, Iba-1 and cytokine was stained from the brains of mice sacrificed at 24 and 48 h after kidney IR injury. The STR, VM, HIP, and CTX regions were stained with Iba-1/IL-6 and Iba-1/TNF-α. We confirmed the colocalization of activated Iba-1 and cytokines, and we also confirmed that the activity of Iba-1 and cytokines increased in the 24 and 48 h groups, compared to the Sham group. Additionally, the colocalization of activated neutrophils and cytokines was analyzed through IF stain. STR, VM, HIP, and CTX regions were stained with Ly-6B.2 (neutrophil maker)/IL-6 and Ly-6B.2/TNF-α. We confirmed the colocalization of activated neutrophils and cytokines, and we also confirmed that the activity of neutrophils and cytokines increased in the 24 and 48 h groups, compared to the Sham group (Supplementary Figures S2–S5).

## 4. Discussion

Acute kidney injury (AKI) refers to a clinical syndrome characterized by a quick decrease in renal excretory function, with the accumulation of products of nitrogen metabolism [25]. Lung injury [26], cardiac failure [27], type 1 hepatorenal syndrome [28], and adverse neurological effects, including ‘uremic encephalopathy’ [29,30], may be caused by AKI. This is constantly linked to an increased production of inflammatory cytokines. Lung-associated systemic inflammatory patterns have been observed in AKI mechanisms [31]. AKI-induced acute heart failure has been shown to affect the kidneys through humoral and immune-mediated pathways and through hemodynamic mechanisms [32]. Hepatic failure is understood to be caused by increased neutrophil infiltration, vascular congestion, and vascular permeability after AKI [9,33,34]. However, the pathophysiology of the CNS is not well-understood. In this study, the physiology of astrocytes and microglia in the brain was investigated through kidney IR-mediated AKI status. For this effect to occur, BBB disruption is a pre-condition. The BBB prevents harmful substances from entering into the brain [22,35]. However, the integrity of the BBB may be altered by neurodegenerative diseases and neuroinflammatory conditions [22]. According to Liu’s study, AKI causes BBB disruption [12]. As shown in the results of the Evans blue permeability test, kidney IR disrupted the BBB at 24 and 48 h. This suggests the potential for peripheral inflammatory cytokines to attack the BBB. Moreover, BBB disruption can act as a trigger for immune responses in the brain.

The accumulation of cytokines in the brain directly causes neuron death [36] and changes the brain immune activity [37]. Therefore, this research team measured the neuronal toxicity and immune response in the region responsible for specific functions in the brain. First, the striatum is an area of the brain involved in motor control and cognitive functions [38,39,40,41]. The role that the striatum plays in motor control is evidenced by the fact that striatal disorders are observed in neurological disorders related to motor control, such as Parkinson’s disease, Huntington’s disease, and Tourette’s disease [42,43]. The VM, which includes two midbrain dopaminergic nuclei, the substantia nigra and the ventral tegmental areas, is the region in which adaptive behavior is based, from working memory to motor control [44]. VM neurons play a distinct role in major disorders, such as Parkinson’s disease, schizophrenia, drug abuse, and attention deficit hyperactivity disorder (ADHD) [44,45]. Huntington’s disease is primarily caused by striatal atrophy, resulting from a loss of GABAergic spiny projection neurons [46]. The main pathological features of Parkinson’s disease are the progressive loss of midbrain dopaminergic neurons and the intraneuronal accumulation of α-synuclein-enriched protein aggregates [47]. The loss of dopaminergic neurons results in a nigrostriatal dopamine deficiency, which causes many of the motor symptoms associated with Parkinson’s disease [48]. In our results, microglia and astrocytes were activated in the striatum and VM. It was confirmed in the expressions of Iba-1 and GFAP, and the increase in cytokines in the striatum and VM was also confirmed at the mRNA level. These results suggest that dopaminergic neurons may be lost due to the activation of microglia and astrocytes in patients who have suffered kidney IR. It has been found that the AKI environment is a risk factor in patients who are genetically susceptible to brain diseases, such as Parkinson’s disease, or who have underlying diseases.

Second, the hippocampus is responsible for memory and learning [49]. Alzheimer’s disease, temporal lobe epilepsy (TLE), cognitive ageing, post-traumatic stress disorder (PTSD), transient global amnesia, schizophrenia, and depressive and anxiety disorders are implicated in hippocampal formation [50]. The cerebral cortex performs several functions, such as sensory, motor, and language functions [51]. Diseases related to the cerebral cortex exhibit different aspects, according to the limbic, prefrontal, frontal, and temporal lobes, but it is generally stated that they are involved in Alzheimer’s, Parkinson’s, and depression [52,53,54,55,56,57]. Amyloid β-protein dimer, which directly induces tau hyperphosphorylation and neurotic degeneration, is considered a direct cause of Alzheimer’s disease [58]. The clinical features of Alzheimer’s include atrophy and neuronal death of the cortex and hippocampus [58,59,60]. We investigated the direct relationship between neurotoxicity and the neuroimmune response associated with AKI. Our results show that only neuroimmune responses are increased without cytotoxicity. In detail, in our results, the microglia and astrocytes were activated in the hippocampus and cortex in the kidney IR scenario. This was further confirmed in the kidney IR scenario in the expressions of Iba-1 and GFAP and the increase in cytokines in the hippocampus and cortex at the mRNA level. If patients who have suffered kidney IR have a genetic risk for Alzheimer’s-like disease or have an underlying medical condition, there is the suggestion that the activation of microglia and astrocytes may lead to neuronal toxicity in the hippocampus and cerebral cortex.

Activated microglia polarize into the M1-phenotype and M2-phenotype. M1-phenotype microglia induce the production of C1q, TNF-α, and IL1-α, which drive astrocytes to transform into A1-reactive astrocytes. Astrocytes lose their normal functions following inflammation and gain the ability to produce harmful substances that kill neurons [24,61,62]. Furthermore, microglia activation can increase astrocyte activation. A recent study identified two phenotypes of reactive astrocytes, termed “A1,” which is neurotoxic and inflammatory, and “A2”, which is neuroprotective and anti-inflammatory. These reactive astrocyte A1 and A2 subtypes are distinguished by distinct gene expression signatures and their functional properties, wherein A1 reactive astrocytes induce neural death [63]. Neuronal death was not observed in the results of this experiment, but an increase in M1- and A1-related genes was observed 24 and 48 h after kidney IR. We observed the specifically upregulated gene expression by A1-specific and pan-reactive astrocytes in the striatum, VM, hippocampus, and cortex in the kidney IR scenario.

There is no direct damage to neurons as a result of brief exposure to AKI, and immune cell activity that can stimulate neurons for a long period or a short period is the main factor. Microglia and astrocyte activation was observed without cytotoxicity in all brain regions. However, there seemed to be no significant difference in the activity of immune cells in the different brain regions.

## 5. Conclusions

The present study evaluated the influence of kidney IR injury on the activation of microglia and astrocytes in brain regions, including the olfactory bulb (OB), the cerebellum (CB), and the brain stem (BS). The results showed that both glia cells were activated by kidney IR. The results of this study confirmed that kidney IR injury activates brain microglia and astrocytes for up to 48 h. Consistently, kidney IR injury induced BBB disruption for up to 48 h in a mouse model. There was increased glial cell activation activity in the whole brain at 24 and 48 h after IR. Cytokine secretion from the microglia and astrocyte toxic type A1 increased at 24 and 48 h after reperfusion. No neuronal cell death was observed in the brain after IR. Furthermore, at 72 h, all inflammatory indicators decreased and returned to normal (Supplementary Figures S6 and S7). However, clinically, when multiple AKI occurs or immune cells induced by kidney injury migrate through peripheral blood vessels and continuously attack the BBB, neuronal cell death is considered inevitable. This provides the first valuable information showing the pathologic aspects of the brain in AKI environments.

## Figures and Tables

**Table 1:** List and sequences of qPCR primers.

Genes	Forward Primer	Reverse Primer
*C1q*	TCTGCACTGTACCCGGCTA	CCCTGGTAAATGTGACCCTTTT
*Il1a*	GCACCTTACACCTACCAGAGT	AAACTTCTGCCTGACGAGCTT
*Il1β*	GCAACTGTTCCTGAACTCAACT	ATCTTTTGGGGTCCGTCAACT
*Tnfa*	CCCTCACACTCAGATCATCTTCT	GCTACGACGTGGGCTACAG
*Il6*	TAGTCCTTCCTACCCCAATTTCC	TTGGTCCTTAGCCACTCCTTC
*C3*	CCAGCTCCCCATTAGCTCTG	GCACTTGCCTCTTTAGGAAGTC
*Steap4*	CCCGAATCGTGTCTTTCCTA	GGCCTGAGTAATGGTTGCAT
*S1pr3*	AAGCCTAGCGGGAGAGAAAC	TCAGGGAACAATTGGGAGAG
*Hspb1*	GACATGAGCAGTCGGATTGA	GGATGGGGTGTAGGGGTACT
*Cxcl10*	CCCACGTGTTGAGATCATTG	CACTGGGTAAAGGGGAGTGA
*Osmr*	GTGAAGGACCCAAAGCATGT	GCCTAATACCTGGTGCGTGT
*Cp*	TGTGATGGGAATGGGCAATGA	AGTGTATAGAGGATGTTCCAGGTCA
*Serpinga 3n*	CCTGGAGGATGTCCTTTCAA	TTATCAGGAAAGGCCGGATTG
*Aspg*	GCTGCTGGCCATTTACACTG	GTGGGCCTGTGCATACTCTT
*Timp1*	AGTGATTTCCCCGCCAACTC	GGGGCCATCATCATGGTATCTGC
*Cd44*	ACCTTGGCCACCACTCCTAA	GCAGTAGGCTGAAGGGTTGT
*Gfap*	AGAAAGGTTGAATCGCTGGA	CGGCGATAGTCGTTAGCTTC
*H2-T23*	GGACCCGCGAATGACATAGC	GCACCTCAGGGTGACTTCAT
*Serping 1*	ACAGCCCCCTCTGAATTCTT	GGATGCTCTCCAAGTTGCTC
*H2-D1*	TCCGAGATTGTAAAGCGTGAAGA	ACAGGGCAGTGCAGGGATAG
*Ggta1*	GTGAACAGCATGAGGGGTTT	GTTTTGTTGCCTCTGGGTGT
*Ligp1*	GGGGCAATAGCTCATTGGTA	ACCTCGAAGACATCCCCTTT
*Gbp2*	GGGGTCACTGTCTCTGACCACT	GGGAAACCTGGGATGAGATT
*Fbln5*	CTTCAGATGCAAGCAACAA	AGGCAGTGTCAGAGGCCTTA
*Ugt1a*	CCTATGGGTCACTTGCCACT	AAAACCATGTTGGGCATGAT
*Fkbp5*	TATGCTTATGGCTCGGCTGG	CAGCCTTCCAGGTGGACTTT
*Psmb8*	CAGTCCTGAAGAGGCCTACG	CACTTTCACCCAACCGTCTT
*Srgn*	GCAAGGTTATCCTGCTCGGA	TGGGAGGGCCGATGTTATTG
*Clcf1*	CTTCAATCCTCCTCCTCGACTGG	TACGTCGGAGTTCAGCTGTG
*Ptx3*	AACAAGCTCTGTTGCCCATT	TCCCAAATGGAACATTGGAT
*S100a10*	CCTCTGGCTGTGGACAAAAT	CTGCTCACAAGAAGCAGTGG
*Cd109*	CACAGTCGGGAGCCCTAAAG	GCAGCGATTTCGATGTCCAC
*Ptgs2*	GCTGTACAAGCAGTGGCAAA	CCCCAAAGATAGCATCTGGA
*Emp1*	GAGACACTGGCCAGAAAAGC	TAAAAGGCAAGGGAATGCAC
*Slc10a6*	GCTTCGGTGGTATGATGCTT	CCACAGGCTTTTCTGGTGAT
*Tm4sf1*	GCCCAAGCATATTGTGGAGT	AGGGTAGGATGTGGCACAAG
*Cd14*	GGACTGATCTCAGCCCTCTG	GCTTCAGCCCAGTGAAAGAC
*Tgm1*	CTGTTGGTCCCGTCCCAAA	GGACCTTCCATTGTGCCTGG
*Sphk1*	GATGCATGAGGTGGTGAATG	TGCTCGTACCCAGCATAGTG

## Data Availability

The data in support of the findings of this study are available from the corresponding author upon reasonable request.

## Supplementary Materials

The following supporting information can be downloaded at: https://www.mdpi.com/article/10.3390/biomedicines10112993/s1, supplementary data.
