# Supplementary material for: Effect of Renal Ischemia Reperfusion on Brain Neuroinflammation
Source: Biomedicines. 2022 Nov 21;10(11):2993. doi: 10.3390/biomedicines10112993 (PMC9687457; doi:10.3390/biomedicines10112993)
Supplement: Supplementary file 1 [file biomedicines-10-02993-s001.zip › biomedicines-2034576-supplementary.pdf]

Supplementary Data S1

Table S1. Striatum, inflammatory cytokine

	Cytokines	Sham	IR 24 h	IR 48 h	Sham & IR 24 h	Sham & IR 48 h
mRNA	c1q	1	2.98±0.44	2.46±017	*	n.s.
	il-1α	1	3.28±0.26	3.91±0.46	**	##
	il-1 β	1	2.13±0.16	2.91±0.25	n.s.	#
	tnfα	1	4.15±0.64	3.62±0.51	****	###
	IL-6	1	4.54±0.70	4.87±0.99	****	####

Table S2. Striatum, Astrocytes

mRNA		Sham	IR 24 h	IR 48 h	Sham & IR 24 h	Sham & IR 48 h
PAN	steap4	1	3.43±0.36	4.8±0.73	****	####
	s1pr3	1	2.14±0.12	2.59±0.27	n.s.	#
	hspb1	1	1.08±0.05	0.81±0.11	n.s.	n.s.
	cxcl10	1	5.13±0.84	6.17±0.54	****	####
	osmr	1	2.49±0.08	2.96±0.19	n.s.	##
	cp	1	2.88±0.19	4.86±0.98	**	####
	serpina3n	1	1.90±0.20	3.20±0.29	n.s.	###
	aspg	1	3.07±0.42	2.92±0.12	**	#
	gfap	1	3.48±0.22	5.63±0.35	****	####
mRNA		Sham	IR 24 h	IR 48 h	Sham &I R 24 h	Sham & IR 48 h
A1	H2-T23	1	3.08±0.34	7.85±0.33	****	####
	Serping1	1	1.14±0.15	1.01±0.15	n.s.	n.s.
	H2-D1	1	3.21±0.11	4.94±0.43	****	####
	Ggta1	1	3.45±0.31	4.33±0.99	****	####
	Ligp1	1	4.56±0.34	4.05±0.44	****	####
	Gbp2	1	1.22±0.07	0.69±0.07	n.s.	n.s.
	Fbln5	1	4.11±0.39	6.72±0.92	****	####
	Ugt1a	1	0.24±0.05	0.56±0.11	n.s.	n.s.
	Fkbp5	1	2.42±0.29	2.94±0.31	*	####
	Psmb8	1	0.72±0.06	1.12±0.05	n.s.	n.s.
	Srgn	1	2.18±0.21	3.76±0.29	n.s.	#
mRNA		Sham	IR 24 h	IR 48 h	Sham & IR 24 h	Sham & IR 48 h
A2	Clcf1	1	0.92±0.09	0.78±0.12	n.s.	n.s.
	Ptx3	1	1.60±0.11	0.87±0.08	n.s.	n.s.
	S100a10	1	1.19±	1.04±0.11	n.s.	n.s.
	Cd109	1	2.13±0.17	1.28±0.11	****	n.s.
	Ptgs2	1	0.80±0.07	1.04±0.12	n.s.	n.s.
	Emp1	1	1.91±0.11	1.87±0.10	***	##
	Slc10a6	1	2.89±0.18	0.47±0.11	****	n.s.
	Tm4sf1	1	1.10±0.15	1.97±0.17	n.s.	###

	d14	1	1.04±0.02	1.04±0.06	n.s.	n.s.

Table S3. V.M Inflammatory cytokines

Cytokines	Sham	IR 24 h	IR 48 h	Sham & IR 24h	Sham & IR 48h
Clq	1	4.7±0.30	3.44±0.51	****	###
IL-1α	1	4.09±0.49	4.40±0.38	****	####
IL-1β	1	5.51±0.82	2.62±0.12	****	n.s
TNF-α	1	1.98±0.08	2.88±0.35	n.s	#
IL-6	1	3.17±0.62	4.53±0.44	**	####

Table S4.V.M Astrocytes

mRNA		Sham	IR 24 h	IR 48 h	Sham &I R 24h	Sham & IR 48h
PAN	steap4	1	2.46±0.16	3.14±0.07	*	####
	s1pr3	1	4.86±0.45	3.26±0.06	****	####
	Timp1	1	2.18±0.24	1.38±0.06	n.s.	n.s.
	hspb1	1	6.76±0.63	3.79±0.78	****	####
	cxcl10	1	3.11±0.33	3.21±0.06	****	####
	cd44	1	1.37±0.24	1.39±0.14	n.s.	n.s.
	osmr	1	3.26±0.45	1.69±0.14	****	n.s.
	cp	1	1.73±0.17	1.20±0.05	n.s.	n.s.
	serpina3n	1	2.90±0.17	1.26±0.18	***	n.s.
	aspg	1	3.29±0.74	5.21±0.10	****	####
	gfap	1	2.75±0.47	5.64±0.87	**	####
A1		Sham	IR 24 h	IR 48 h	Sham & IR 24h	Sham & IR 48h
	H2-T23	1	1.46±0.12	0.94±0.13	n.s.	n.s.
	Serping1	1	0.96±0.17	0.61±0.08	n.s.	n.s.
	H2-D1	1	5.79±0.81	3.36±0.44	****	####
	Ggta1	1	2.73±0.40	4.41±0.79	**	####
	Ligp1	1	0.51±0.03	0.85±0.06	n.s.	n.s.
	Gbp2	1	1.13±0.04	1.51±0.12	n.s.	n.s.
	Fbln5	1	1.05±0.08	1.99±0.36	n.s.	n.s.
	Ugt1a	1	0.26±0.06	0.55±0.04	n.s.	n.s.
	Fkbp5	1	4.07±0.23	5.98±0.49	****	####
	Psmb8	1	5.82±0.44	2.78±0.60	****	##
	Srgn	1	4.60±0.56	4.30±0.68	****	####
A2		Sham	IR 24 h	IR 48 h	Sham & IR 24h	Sham & IR 48h
	Clcf1	1	0.35±0.11	0.8±0.16	n.s	n.s.
	Tgm1	1	2.00±0.19	1.23±0.005	n.s	n.s.
	Ptx3	1	0.70±0.06	0.78±0.09	n..	n.s.
	S100a10	1	0.86±0.09	1.46±0.13	n.s.	n.s.
	Sphk1	1	0.85±0.09	0.81±0.07	n.s.	n.s.
	Cd109	1	0.43±0.05	1.62±0.20	n.s.	n.s.

	Ptgs2	1	0.78±0.10	1.19±0.18	n.s.	n.s.
	Emp1	1	0.26±0.02	1.89±0.16	n.s.	n.s.
	Slc10a6	1	0.48±0.06	1.06±0.14	n.s	n.s.
	Tm4sf1	1	1.27±0.006	1.11±0.07	n.s	n.s.
	Cd14	1	0.23±0.03	0.85±0.07	n.s	n.s.

Table S5. HIP, Cytokines

	Cytokines	Sham	IR 24 h	IR 48 h	Sham & IR 24h	Sham & IR 48h
mRNA	Clq	1	2.04±0.21	4.24±0.24	n.s.	####
	IL-1α	1	4.98±0.63	5.45±0.45	****	####
	IL-1β	1	1.65±0.41	1.05±0.12	n.s.	n.s.
	TNF-α	1	3.33±0.19	8.73±0.83	***	####
	IL-6	1	2.66±0.28	4.05±0.74	n.s.	####

Table S6. HIP, Astrocytes

	mRNA	Sham	IR 24 h	IR 48 h	Sham & IR 24 h	Sham & IR 48 h
PAN	s1pr3	1	1.77±0.09	2.14±0.15	n.s.	***
	Timp1	1	1.08±0.13	1.21±0.05	n.s.	n.s.
	hspb1	1	0.42±0.07	0.60±0.06	n.s.	n.s.
	cxcl10	1	3.27±0.11	4.37±0.31	****	####
	osmr	1	1.60±0.07	3.09±0.14	n.s.	####
	cp	1	2.35±0.08	4.30±0.15	****	####
	serpina3n	1	1.80±0.06	1.75±0.06	*	n.s.
	aspg	1	3.97±0.42	3.65±0.51	****	####
	gfap	1	2.23±0.13	3.44±0.42	****	####
A1	H2-T23	1	1.59±0.16	3.20±0.27	n.s.	####
	Serping1	1	2.34±0.12	4.31±0.56	*	####
	H2-D1	1	1.11±0.18	1.32±0.13	n.s.	n.s.
	Ggta1	1	2.19±0.16	3.63±0.35	n.s.	####
	Ligp1	1	3.19±0.29	3.87±0.34	****	####
	Gbp2	1	2.14±0.12	3.41±0.14	n.s.	####
	Fbln5	1	3.23±0.34	3.77±0.38	****	####
	Ugt1a	1	1.71±0.14	3.28±0.50	n.s.	####
	Fkbp5	1	1.71±0.43	1.49±0.16	n.s.	n.s.
	Psmb8	1	0.97±0.14	0.86±0.13	n.s.	n.s.
	Srgn	1	2.41±0.25	4.73±0.77	**	####
A2	Clcf1	1	1.00±0.04	0.59±0.06	n.s.	n.s.
	Ptx3	1	2.03±0.16	1.16±0.13	n.s.	n.s.
	S100a10	1	1.24±0.05	1.36±0.11	n.s.	n.s.

	Cd109	1	3.48±0.56	1.72±0.16	****	n.s.
	Ptgs2	1	1.00±0.09	1.11±0.14	n.s.	n.s.
	Emp1	1	2.25±0.23	2.00±0.17	*	n.s.
	Slc10a6	1	1.56±0.45	0.58±0.15	n.s.	n.s.
	Tm4sf1	1	1.16±0.04	2.21±0.42	n.s.	#
	Cd14	1	1.84±0.25	1.95±0.17	n.s.	n.s.

Table S7. Cytokines Cortex

	cytokines	Sham	IR 24 h	IR 48 h	Sham & IR 24 h	Sham & IR 48 h
mRNA	Clq	1	2.45±0.06	4.14±0.50	n.s.	####
	IL-1α	1	4.76±0.68	2.88±0.08	****	#
	TNF-α	1	3.71±0.61	2.23±0.07	****	n.s.
	C3	1	4.04±0.22	2.69±0.54	****	#
	IL-6	1	5.95±0.80	4.17±0.76	****	####

Table S8. Cortex Astrocytes

	mRNA	Sham	IR 24 h	IR 48 h	Sham & IR 24h	Sham & IR 48h
PAN	steap4	1	5.04±0.72	4.44±0.40	****	####
	s1pr3	1	1.57±0.02	2.39±0.15	n.s.	#
	Timp1	1	2.17±10.04	2.24±0.04	n.s.	n.s.
	hspb1	1	2.32±0.16	4.07±0.12	n.s.	####
	cxcl10	1	3.81±0.28	4.39±0.46	****	####
	cd44	1	1.44±0.03	1.21±0.04	n.s.	n.s.
	osmr	1	1.62±0.02	2.26±0.05	n.s.	n.s.
	cp	1	7.75±0.27	6.67±1.03	****	####
	serpina3n	1	1.64±0.07	1.79±0.01	n.s.	n.s.
	aspg	1	5.46±0.34	3.36±0.08	****	####
	gfap	1	5.54±0.92	4.82±0.52	****	####
A1	H2-T23	1	3.29±0.61	2.67±0.38	***	##
	Serping1	1	0.76±0.02	1.70±0.2	n.s.	n.s.
	H2-D1	1	4.09±0.09	2.43±0.06	****	**
	Ggta1	1	4.82±0.71	2.20±0.04	****	n.s.
	Ligp1	1	0.75±0.04	1.15±0.02	n.s.	n.s.
	Gbp2	1	0.78±0.03	0.68±0.02	n.s.	n.s.

	Fbln5	1	4.27±0.12	6.25±0.93	****	####
	Ugt1a	1	3.32±0.07	3.54±0.05	****	####
	Fkbp5	1	3.61±0.18	2.38±0.07	****	##
	Psmb8	1	1.09±0.02	1.18±0.03	n.s.	n.s.
	Srgn	1	4.18±0.85	3.13±0.0	****	####
A2	Clcf1	1	0.98±0.02	1.30±0.0.4	n.s.	n.s.
	Tgm1	1	2.11±0.04	1.62±0.19	***	n.s.
	Ptx3	1	0.62±0.02	1.07±0.07	n.s.	n.s.
	S100a10	1	0.72±0.03	1.32±0.04	n.s.	n.s.
	Sphk1	1	4.53±0.08	1.59±0.10	****	n.s.
	Cd109	1	0.63±0.04	0.77±0.03	n.s.	n.s.
	Ptgs2	1	0.54±0.03	0.49±0.01	n.s.	n.s.
	Emp1	1	1.12±0.03	1.63±0.02	n.s.	n.s.
	Slc10a6	1	1.33±0.03	4.49±0.05	n.s.	####
	Tm4sf1	1	0.86±0.02	1.56±0.03	n.s.	n.s.
	Cd14	1	0.77±0.05	0.65±0.02	n.s.	n.s.

Renal IR induced markers of proimflammatory cytokines and astrocytes, center values represent mean ± SEM (n=9 independent experiments). Two-way ANOVA followed by Bonferroni multiple comparison test was used for statistical significance. **p* <0.05, ***p* <0.01, ****p* <0.001, *****p* <0.0001 vs. I/R24 h with Sham, #*P*< 0.05, ##*p* <0.01, ###*p* <0.001, ####*p* <0.0001 vs. I/R 48 h with sham.

Supplementary figures

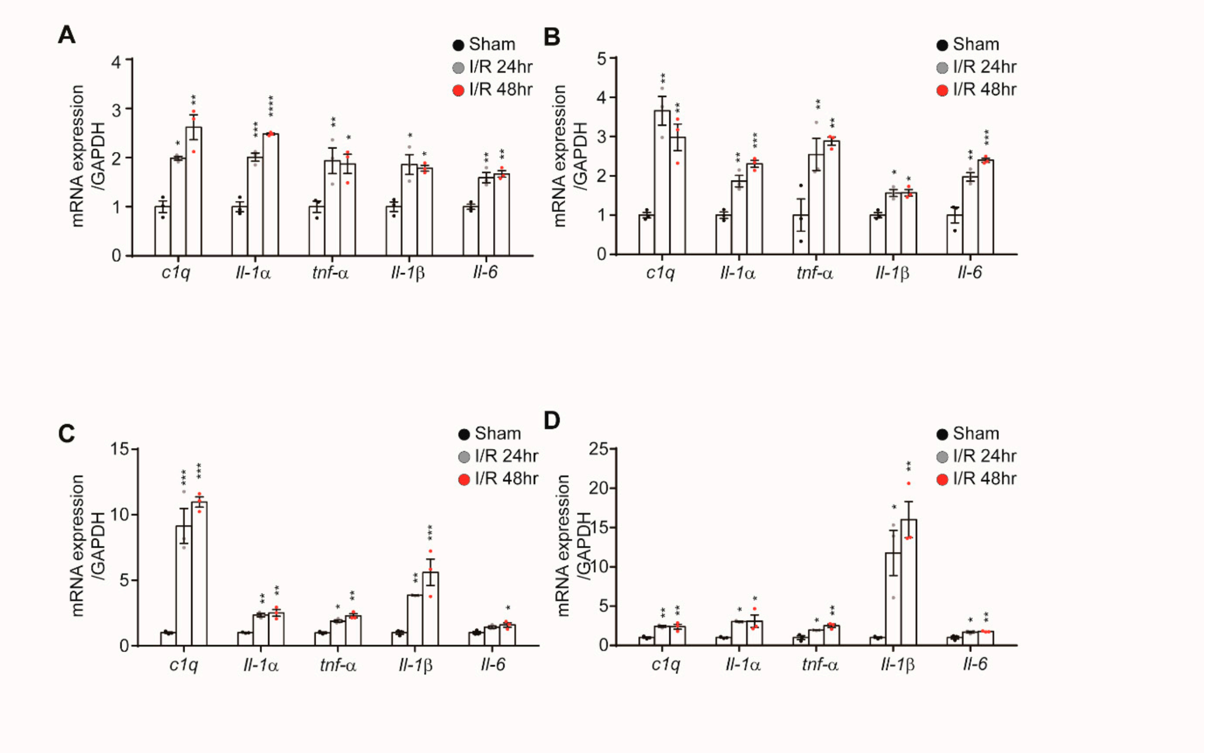

Supplementary Figure S1. The expression of inflammatory mediators (*C1q, Il1a, Il1b, Tnfa,* and *Il6*) were determined using quantitative qPCR analysis in RNA samples of stratum brain tissues. (A) Cytokine mRNA expression of STR region. (B) Cytokine mRNA expression of VM region. (C) Cytokine mRNA expression of HIP region. (D) Cytokine mRNA expression of CTX region. Values are represented as mean ± S.E.M of independent mice. *p<0.05, **p<0.01, ***p<0.001, ****p<0.0001 significant compare with sham group.

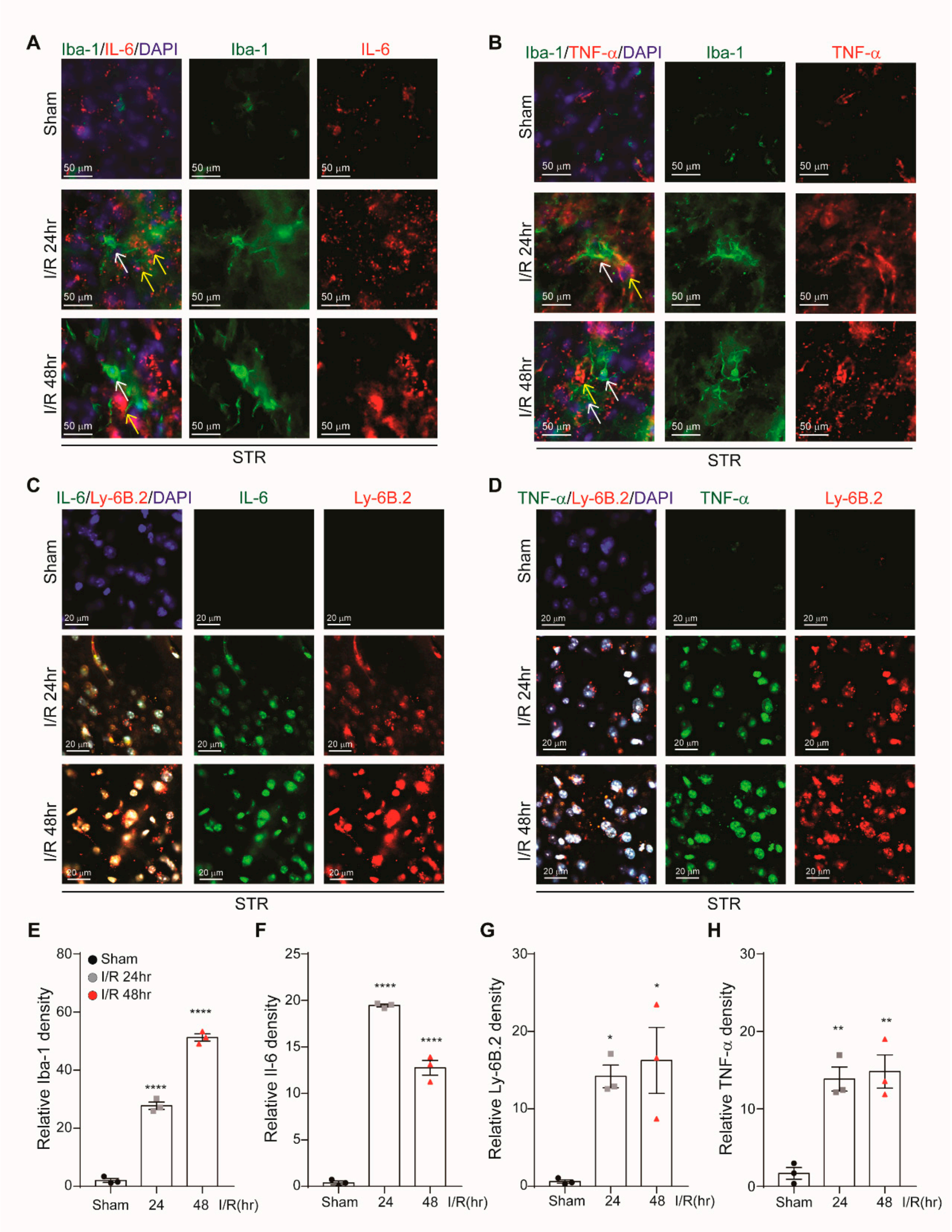

Supplementary Figure S2. Images and graphs of immunofluorescent (IF) colocalization of activated microglial and the cytokines in STR region. (A) Frozen brain tissues were dissected and then incubated with anti-Iba-1 and anti-IL-6 antibodies for IF staining (n = 3, biologically independent animals). (B) Frozen brain tissues were dissected and then incubated with anti-Iba-1 and anti-TNF-α antibodies for IF staining (n = 3, biologically independent animals). (C) Frozen brain tissues were dissected and then incubated with anti-IL-6 and anti-Ly-6B.2 antibodies for IF staining (n = 3, biologically independent animals). (D) Frozen brain tissues were dissected and then incubated with anti-IL-6 and anti-Ly-6B.2 antibodies for IF staining. (E-H) Graph of relative Iba-1, Il-6, Ly-6B.2 and TNF-α density, measuring using Image J. Values are represented as mean ± S.E.M of independent mice. *p<0.05, **p<0.01, ***p<0.001, ****p<0.0001 significant compare with sham group.

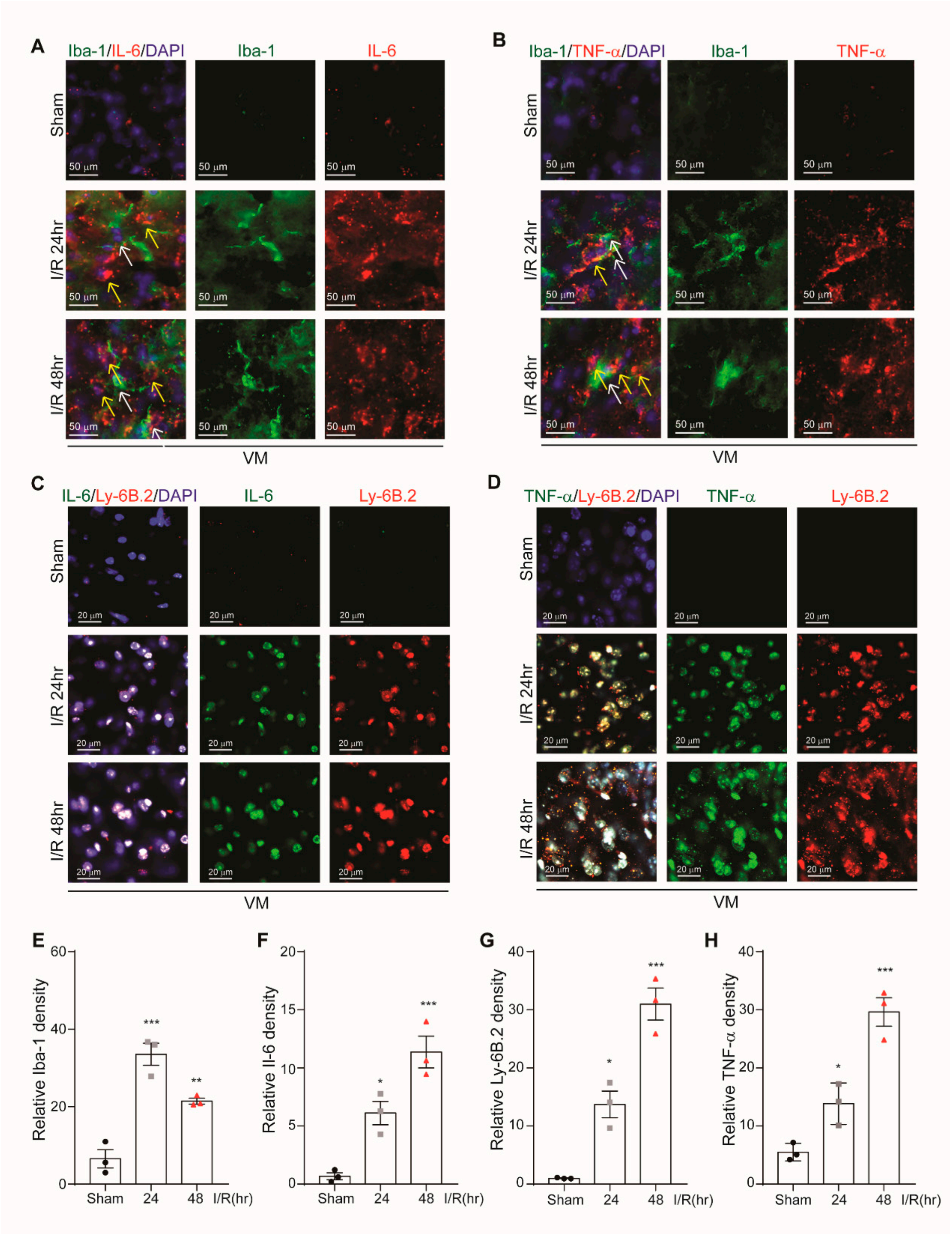

Supplementary Figure S3. Images and graphs of IF colocalization of activated microglial and the cytokines in VM region. (A) Frozen brain tissues were dissected and then incubated with anti-Iba-1 and anti-IL-6 antibodies for IF staining (n = 3, biologically independent animals). (B) Frozen brain tissues were dissected and then incubated with anti-Iba-1 and anti-TNF-α antibodies for IF staining (n = 3, biologically independent animals). (C) Frozen brain tissues were dissected and then incubated with anti-IL-6 and anti-Ly-6B.2 antibodies for IF staining (n = 3, biologically independent animals). (D) Frozen brain tissues were dissected and then incubated with anti-IL-6 and anti-Ly-6B.2 antibodies for IF staining. (E-H) Graph of relative Iba-1, Il-6, Ly-6B.2 and TNF-α density, measuring using Image J. Values are represented as mean ± S.E.M of independent mice. *p<0.05, **p<0.01, ***p<0.001, ****p<0.0001 significant compare with sham group.

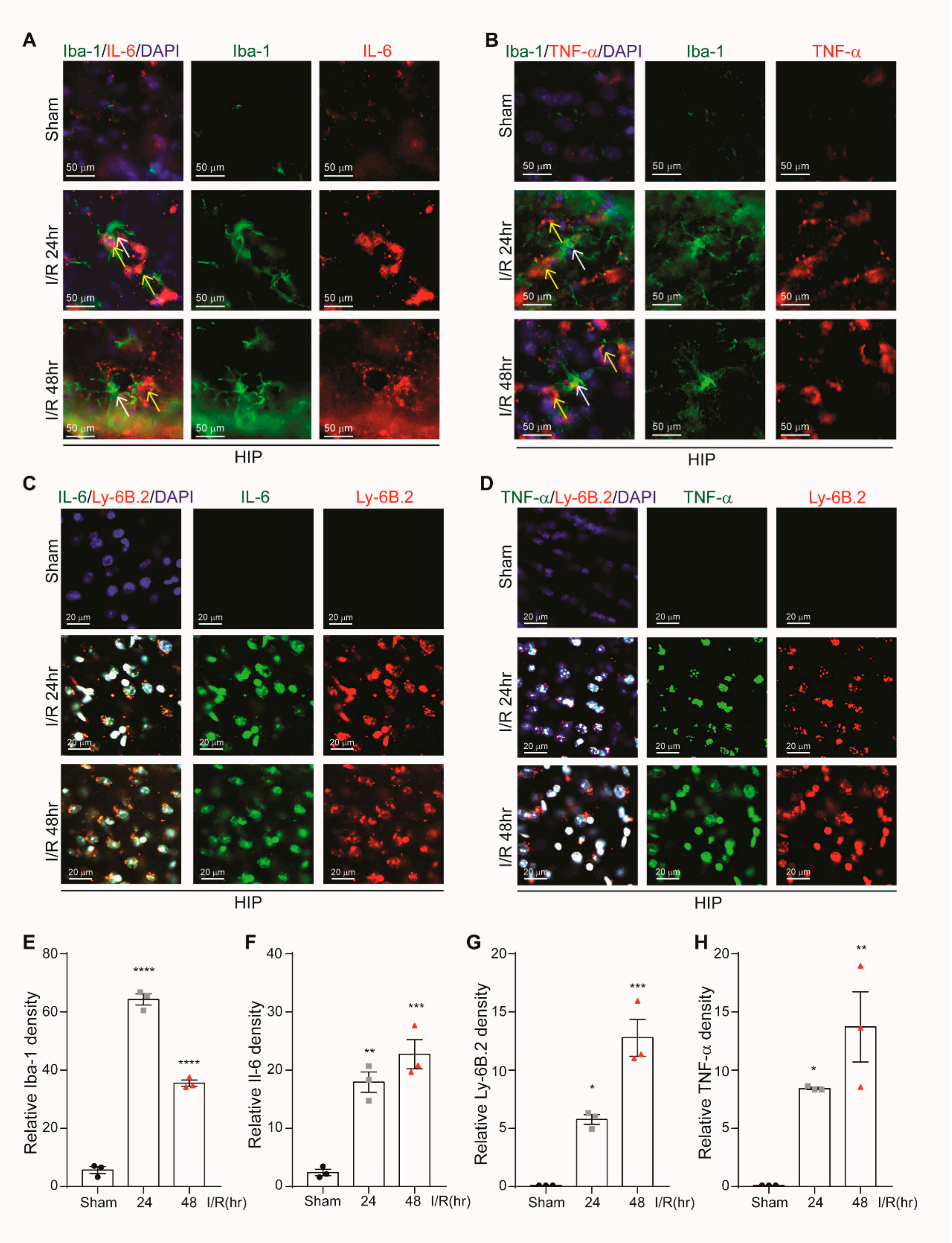

Supplementary Figure S4. Images and graphs of IF colocalization of activated microglial and the cytokines in HIP region. (A) Frozen brain tissues were dissected and then incubated with anti-Iba-1 and anti-IL-6 antibodies for IF staining (n = 3, biologically independent animals). (B) Frozen brain tissues were dissected and then incubated with anti-Iba-1 and anti-TNF-α antibodies for IF staining (n = 3, biologically independent animals). (C) Frozen brain tissues were dissected and then incubated with anti-IL-6 and anti-Ly-6B.2 antibodies for IF staining (n = 3, biologically independent animals). (D) Frozen brain tissues were dissected and then incubated with anti-IL-6 and anti-Ly-6B.2 antibodies for IF staining. (E-H) Graph of relative Iba-1, Il-6, Ly-6B.2 and TNF-α density, measuring using Image J. Values are represented as mean ± S.E.M of independent mice. *p<0.05, **p<0.01, ***p<0.001, ****p<0.0001 significant compare with sham group.

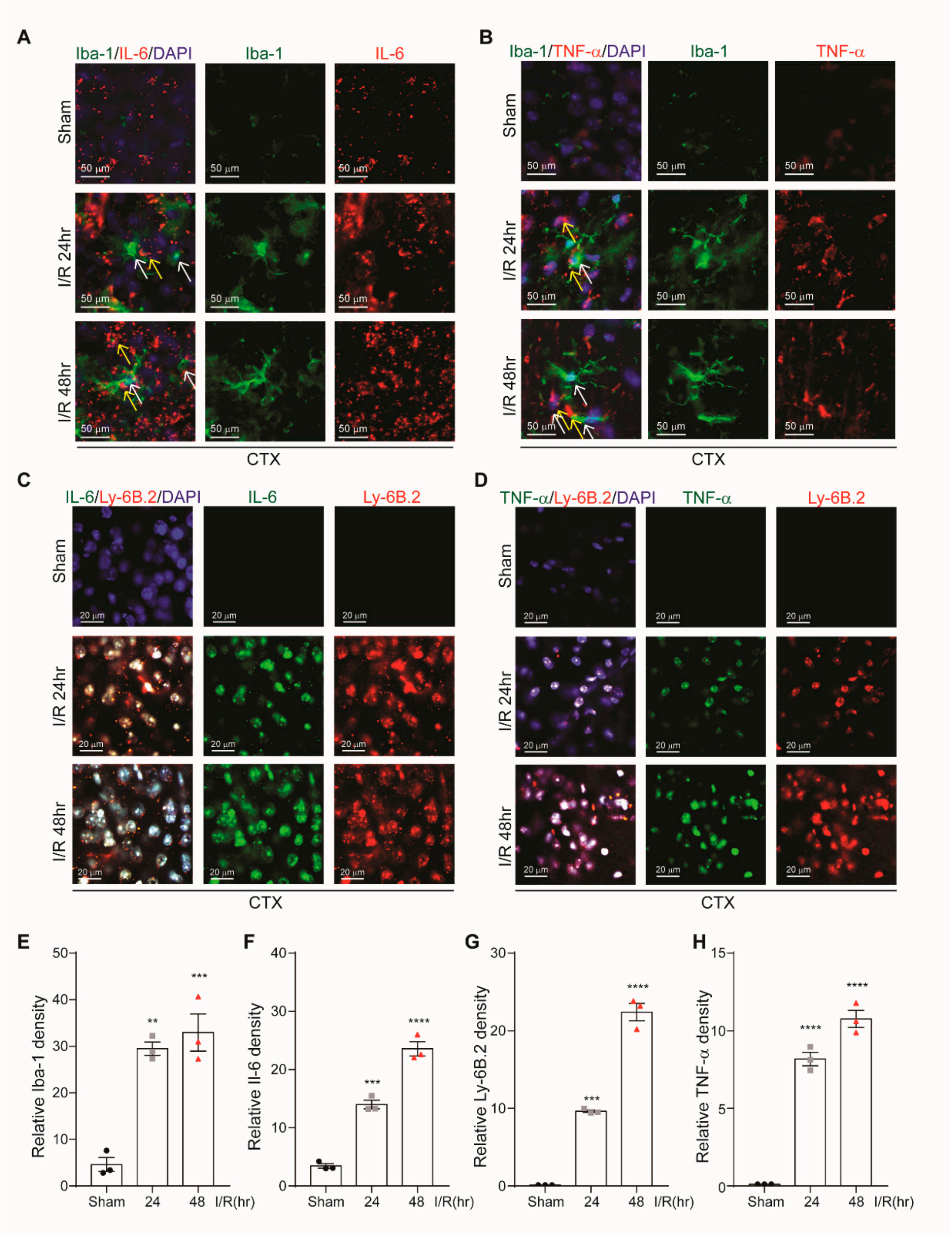

Supplementary Figure S5. Images and graphs of IF colocalization of activated microglial and the cytokines in CTX region. (A) Frozen brain tissues were dissected and then incubated with anti-Iba-1 and anti-IL-6 antibodies for IF staining (n = 3, biologically independent animals). (B) Frozen brain tissues were dissected and then incubated with anti-Iba-1 and anti-TNF-α antibodies for IF staining (n = 3, biologically independent animals). (C) Frozen brain tissues were dissected and then incubated with anti-IL-6 and anti-Ly-6B.2 antibodies for IF staining (n = 3, biologically independent animals). (D) Frozen brain tissues were dissected and then incubated with anti-IL-6 and anti-Ly-6B.2 antibodies for IF staining. (E-H) Graph of relative Iba-1, Il-6, Ly-6B.2 and TNF-α density, measuring using Image J. Values are represented as mean ± S.E.M of independent mice. *p<0.05, **p<0.01, ***p<0.001, ****p<0.0001 significant compare with sham group.

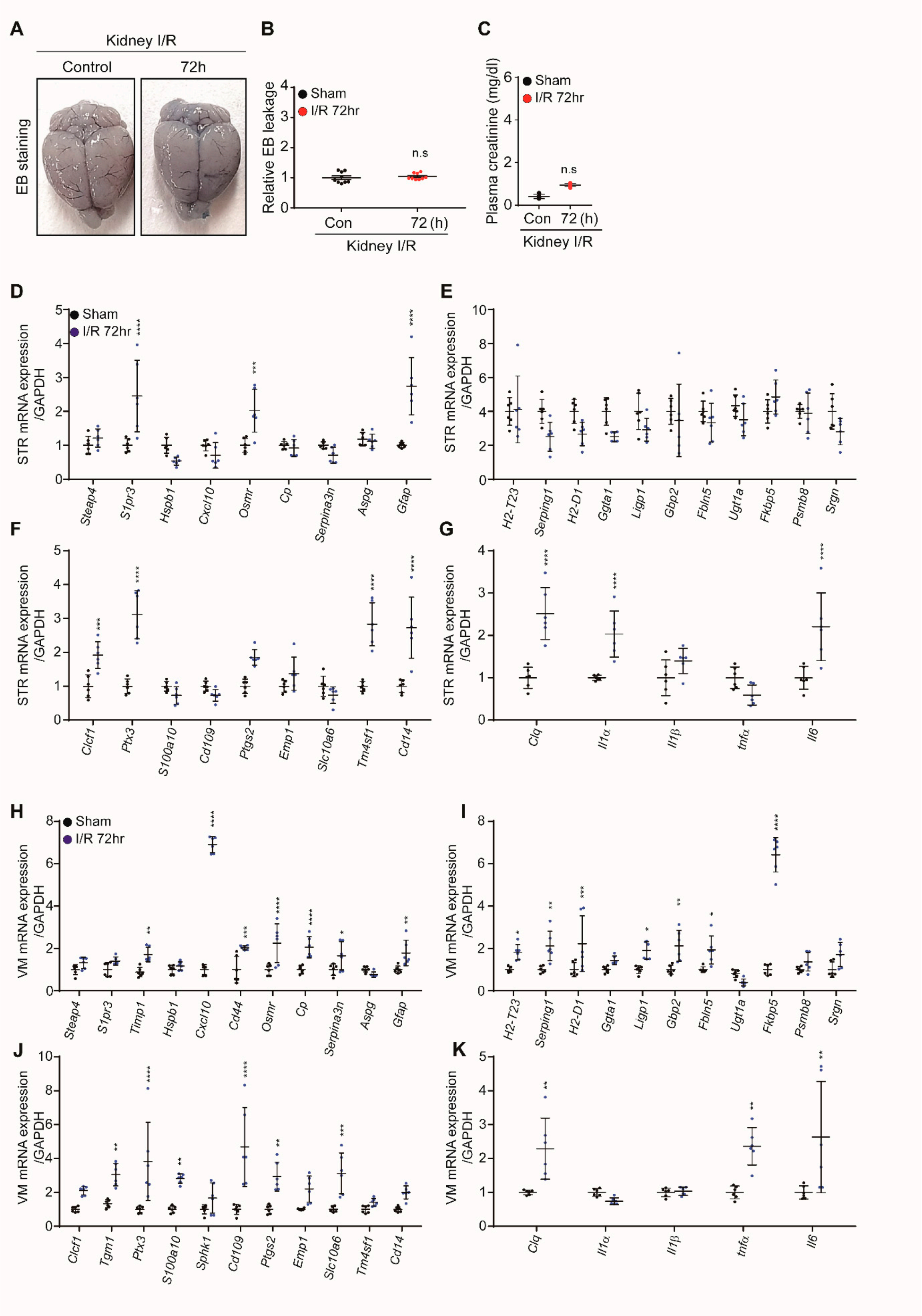

Supplementary Figure S6. Kidney IR injury impaired BBB permeability. (A) C57BL/6 mice sham (control) were operated, mice subjected to 25 min bilateral kidney IR and sacrificed at 72 h after reperfusion. (B) Relative EB

leakage levels in the brain samples. (C) Plasma creatinine level. The data represent the mean ± S.E.M for nine mice from each group $p<0.005$ the significant compared with the sham group. (D-F) The gene markers of astrocytes; PAN-reactive transcripts, A1-specific transcripts, A2-specific transcripts, were examined using quantitative PCR analysis, in STR RNA samples respectively. (G) The expression of inflamma-tory mediators (C1q, Il1a, Il1b, Tnfa and Il6) were determined using quantitative qPCR analysis, in RNA samples of STR tissues. (H-J) The gene markers of astrocytes; PAN-reactive transcripts, A1-specific transcripts, A2-specific transcripts, were examined using quantitative PCR analysis, in VM RNA samples respectively. (K) The expression of inflamma-tory mediators (C1q, Il1a, Il1b, Tnfa and Il6) were determined using quantitative qPCR analysis, in RNA samples of VM tissues. Values are represented as mean ± S.E.M of independent mice. *$p<0.05$, **$p<0.01$, ***$p<0.001$, ****$p<0.0001$ significant compare with sham group.

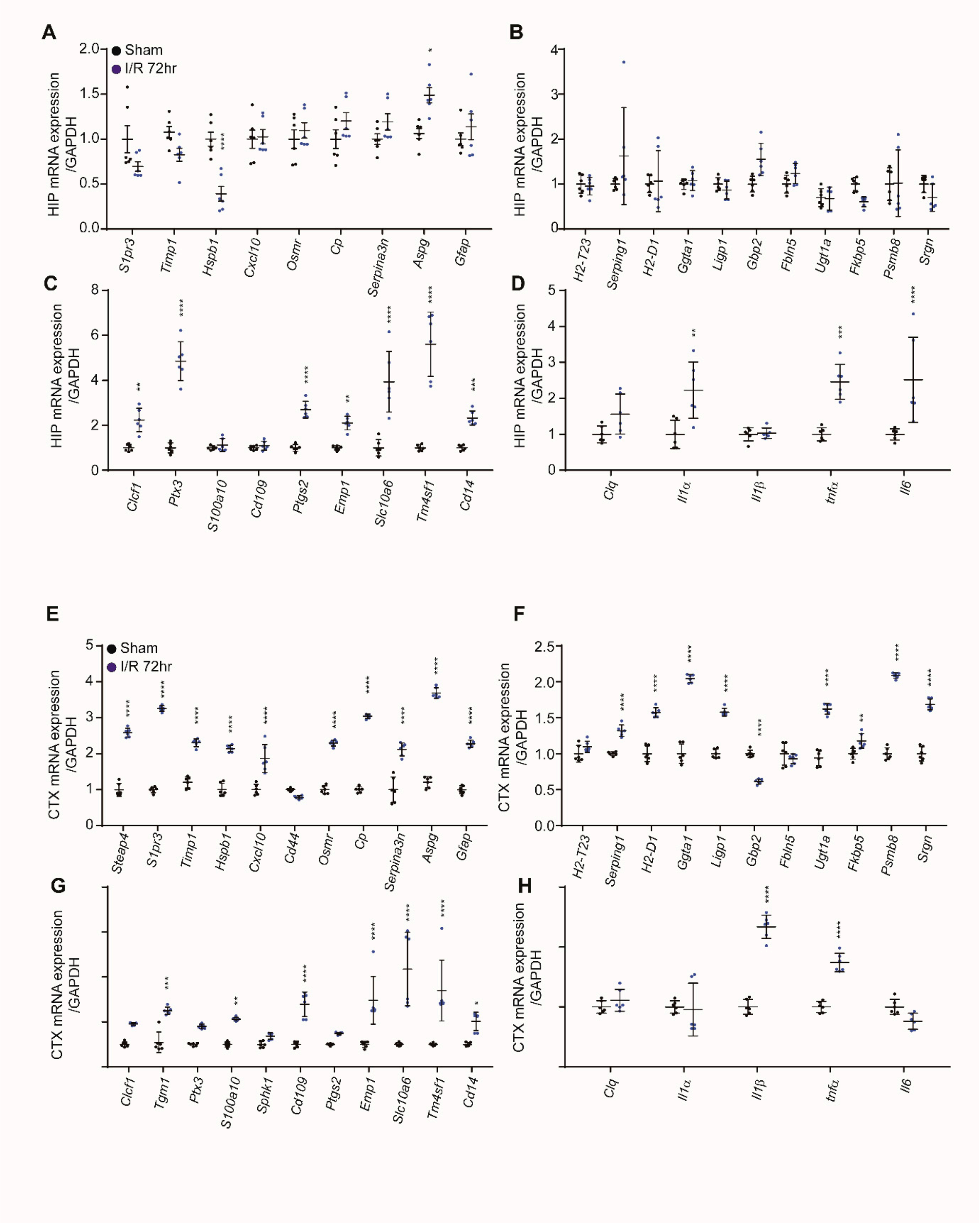

Supplementary Figure S7. Kidney IR injury impaired BBB permeability. (A-C) The gene markers of astrocytes; PAN-reactive transcripts, A1-specific transcripts, A2-specific transcripts, were examined using quantitative PCR analysis, in HIP RNA samples respectively. (D) The expression of inflammatory mediators (C1q, Il1a, Il1b, Tnfa and Il6) were determined using quantitative qPCR analysis, in RNA samples of HIP tissues. (E-G) The gene markers of astrocytes; PAN-reactive transcripts, A1-specific transcripts, A2-specific transcripts, were examined using quantitative PCR analysis, in CTX RNA samples respectively. (H) The expression of inflamma-tory mediators (C1q, Il1a, Il1b, Tnfa and Il6) were determined using quantitative qPCR analysis, in RNA samples of CTX tissues. Values are represented as mean ± S.E.M of independent mice. *p<0.05, **p<0.01, ***p<0.001, ****p<0.0001 significant compare with sham group.
